# Application of Gas‐Phase Electrophoresis (nES GEMMA Instrumentation) in Molecular Weight Determination

**DOI:** 10.1002/jms.5183

**Published:** 2025-10-08

**Authors:** Victor U. Weiss, Wladyslaw W. Szymanski, Martina Marchetti‐Deschmann

**Affiliations:** ^1^ Institute of Chemical Technologies and Analytics TU Wien Vienna Austria; ^2^ Faculty of Physics, Aerosol Physics and Environmental Physics University of Vienna Vienna Austria

**Keywords:** DMA, gas‐phase electrophoresis, molecular weight, nES GEMMA, SMPS

## Abstract

Gas‐phase electrophoresis by means of a nano‐Electrospray Gas‐phase Electrophoretic Mobility Molecular Analyzer (nES GEMMA, also known as e.g., nES DMA, MacroIMS, or LiquiScan ES) separates singly charged, aerosolized (bio‐)nanoparticles at ambient pressure in the gas phase according to the particle electrophoretic mobility (EM) diameter, i.e., an equivalent size related to spherical analytes. Corresponding size spectra in the range of a few to several hundred nanometers in terms of an EM diameter relate particle number concentrations to particle size values. Already shortly after the introduction of the instrument to the community of analytical chemists, its ability to yield particle molecular weight (MW) values based on measured EM diameters applying a corresponding correlation function was described. This first calibration function was solely based on protein monomers, dimers, and larger aggregates. In the following years, it became evident that this correlation function was well suited to calculate MW of globular proteins based on their EM diameters. For other substance classes, significant deviations were observed which ultimately led to the definition of further calibration functions. This manuscript reviews these developments and provides an overview of some key applications of gas‐phase electrophoresis using the nES GEMMA system for MW determination of (bio‐)nanoparticles from initial experiments in the year 1996 to the current day (2025).

## 1 Introduction

According to the recommendations of the European Commission for nanoparticle characterization (2011/696/EU, October 18th, 2011, updated version 2022/C 229/01, June 10th, 2022), nanoparticle materials are defined as matter with “50% or more of particles having a size between 1 nm and 100 nm” for assessing the materials characteristics concerning their impact on health, environment, or safety. In this context, gas‐phase electrophoresis using a nano electrospray gas‐phase electrophoretic mobility molecular analyzer (nES GEMMA) is a competitive analytical technique. Measurements are performed at ambient pressure, opening new characterization avenues and complementing analytical techniques requiring vacuum. The determination of nanoparticle abundance vs. electrophoretic mobility (EM) is fast, and the obtained spectra are rather uncomplicated, making data interpretation relatively straightforward. EM data, in turn, allows an assessment of the MW in the kilo‐ to megadalton range of an analyte in question based directly on instrumental readouts and application of a corresponding correlation function. It is thus the aim of this review to describe the instrumentation and its applicability in analyte molecular weight (MW) determination in detail, from first gas‐phase electrophoresis experiments in 1996 to the current state (2025).

## 2 On the Beginning of Gas‐Phase Electrophoresis

**FIGURE 1:**
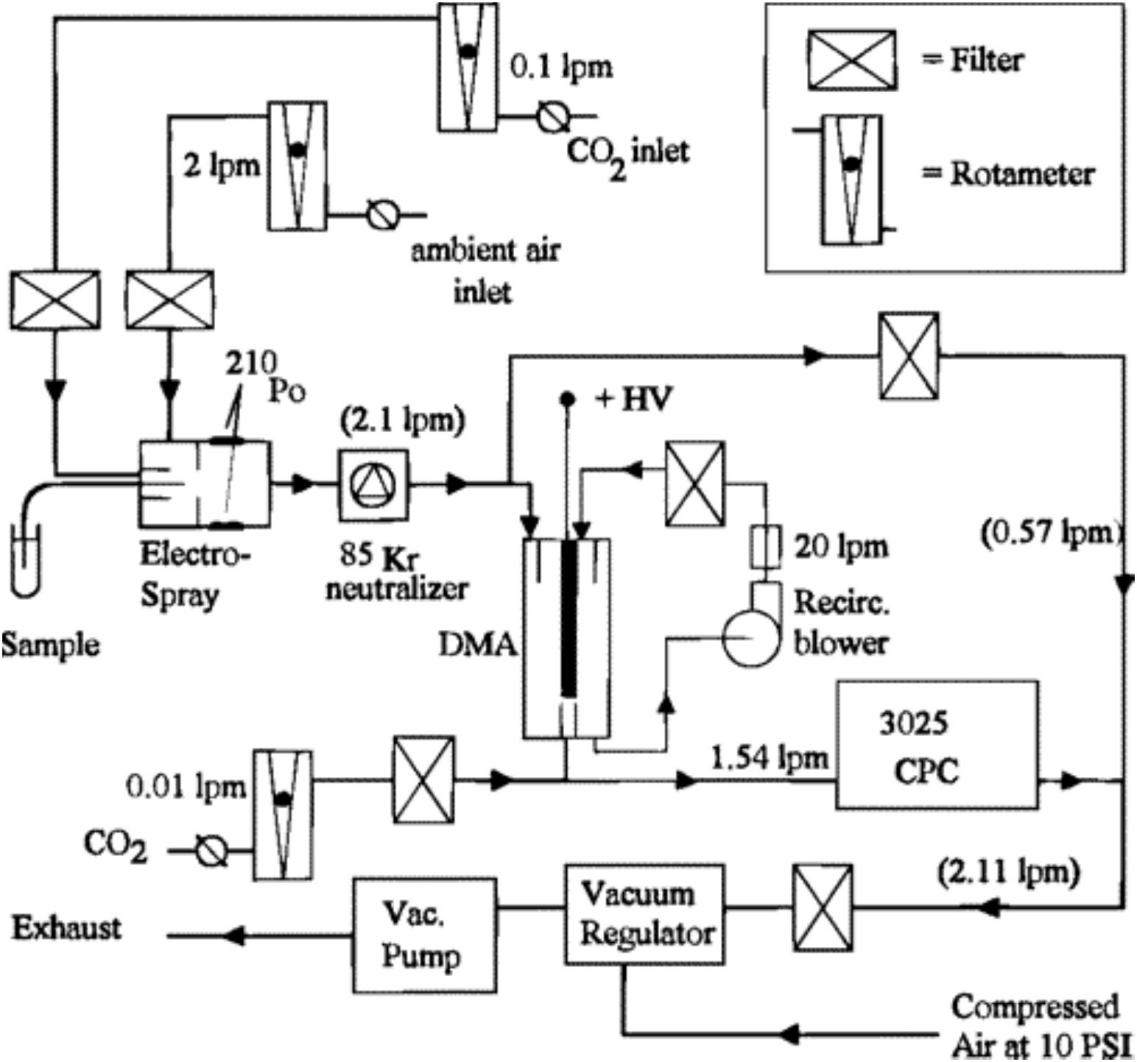
The first description of a nES GEMMA instrumentation taken from Kaufman et al. Reprinted (adapted) with permission from [1]. Copyright 1996 American Chemical Society.

Gas‐phase electrophoresis on a nES GEMMA as a stand‐alone instrumentation was first described by Kaufman et al. in 1996 [1]. In this manuscript, Kaufman and colleagues used (i) a cone‐tipped fused silica capillary for a nES, (ii) a ^210^Po alpha‐particle emitter as a source to obtain a stable bipolar atmosphere in order to charge equilibrate undefined multi‐charged aerosol droplets from the nES process, (iii) a nano differential mobility analyzer (nDMA), and (iv) a n‐butanol‐based ultrafine condensation particle counter (CPC) for number‐based particle detection after separation of the polydisperse aerosol in the nDMA (see Figure 1). Analytes were electrosprayed, followed by drying of droplets and concomitant charge equilibration. Resulting polydisperse aerosol particles were mostly neutral. However, a certain known percentage of single‐charged analytes was also obtained. Separation of the latter occurred in a highly laminar sheath flow of air and a perpendicular tunable electric field. Subsequently, separated particles with known sizes were monitored with a CPC, in which, after a nucleation process induced by separated analytes in a supersaturated n‐butanol atmosphere, a detection process by laser‐light scattering occurred. Proteins ranging from bovine insulin monomers (~5.7 kDa) to bovine thyroglobulin dimers (~1338 kDa) were measured, and resulting EM diameters plotted against analyte MW values. In doing so, the analyte behavior upon transition from the liquid to the gas‐phase was investigated, focusing on protein density values to gather information on the particle folding and orientation in the gas‐phase. Subsequently, this instrumental setup was commercialized by a US‐based company, TSI Inc. (Shoreview, MN, USA). However, it should be noted that the concepts of the individual instrumental parts were known already for some time prior to Kaufman's experiments with globular proteins in 1996.

Electrospray ionization, extensively studied by Fenn and colleagues in the 1980s, enabled the transfer of large, multicharged (bio‐)molecules from the liquid to the gas phase in the field of mass spectrometry; a process called sometimes “teaching elephants to fly” [2, 3]. In 1995, Chen and colleagues (including Stanley Kaufman) had focused on the generation of monodisperse aerosol particles for the purpose of instrument calibration based on ES processes [4]. Charge equilibration in a bipolar atmosphere, on the other hand, was already investigated much earlier e.g., by Liu and Pui [5], or Wiedensohler and Fissan [6]. (For additional information on charge equilibration setups, please refer to “Conclusions and Outlook”.) As electrophoretic separations are usually based on charge and size/shape of analytes, concentration on single‐charged species leads to significantly less complicated spectra as separation is solely based on particle size upon electrophoresis in the gas‐phase.

Likewise, the DMA concept dates back to the 1920s and originates in aerosol studies and atmospheric measurements during that time. In short, two concentric electrodes enable the application of a high, tunable voltage resulting in a defined electric field between them governing the trajectories of charged particles in question in an otherwise particle‐free, highly laminar sheath flow of air. Particle counting enables characterizing aerosol particles in their respective concentration and size. Reviews by Juan de la Mora and colleagues [7] as well as Intra and Tippayawong [8] give an excellent overview of the technique. Ultimately, bringing all these individual concepts together for (bio‐)nanoparticle characterization introduced a novel instrumental arrangement to the analytical community [1]. Following that work, a first nES GEMMA review article was published in 1998 [9].

It is of note that over the years, different instrument names were reported in literature—nES GEMMA, MacroIMS, LiquiScan ES, SMPS, or nES DMA. In terms of surveying literature, this might be sometimes problematic. However, all these instruments—despite different names—share the same overall analytical principle: the determination of particle number concentrations in relation to the applied separation voltage in the DMA unit of the used instrument. This latter number can be correlated with the surface‐dry particle diameter of spherical analytes in the gas phase and is usually referred to as EM diameter or EMD, given in [nm]. In 2012, Guha and colleagues reviewed such an analytical instrument as “rapid, high resolution and accurate size characterization in a multi‐component environment” [10].

## 3 Gas‐Phase Electrophoresis Based MW Determination of Proteins

**FIGURE 2:**
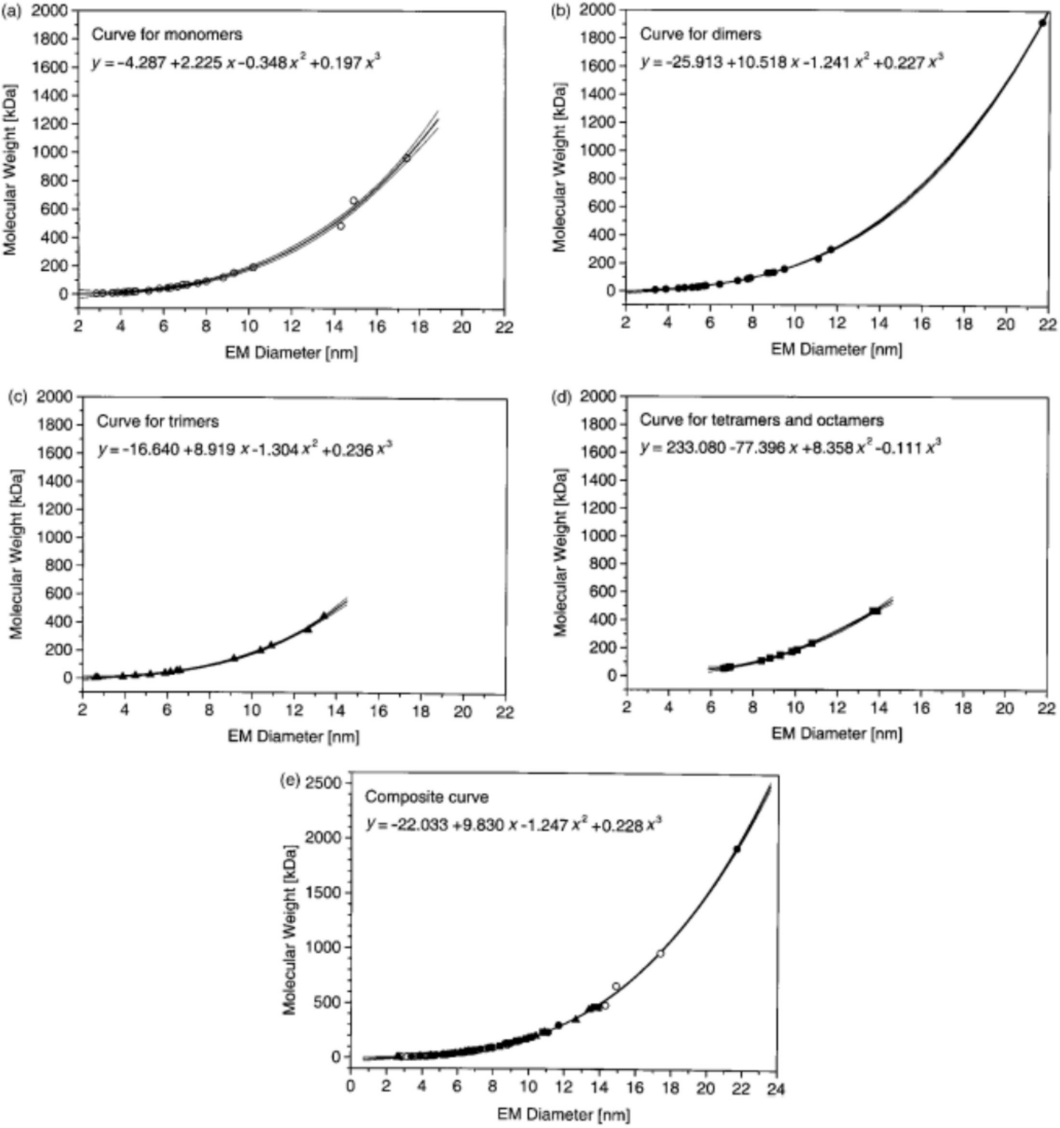
The first MW/EM diameter correlation published for proteins by Bacher et al. in Journal of Mass Spectrometry by John Wiley and Sons in 2001 [11]. Copyright 2001 John Wiley & Sons Ltd., reproduced with permission.

It was soon after the initial publication by Kaufman et al. in 1996 that the applicability of the novel nES GEMMA concept in proteomics studies was investigated by the group around Günter Allmaier. In a seminal manuscript published in 2001 [11], Bacher, Allmaier, and colleagues showed that proteins and protein aggregates of different MW values yielded different EM diameters upon gas‐phase electrophoresis. Influenced by mass spectrometry, the group set up an EM diameter/MW correlation based on gas‐phase electrophoresis data for protein monomers, dimers, trimers, and multimers for the first time (see Figure 2). It was only two decades later that the combination of size exclusion chromatography with another generation of gas‐phase electrophoresis instrumentation demonstrated a slight error in this first correlation: nES‐based protein aggregates appeared to slightly differ from liquid‐phase formed aggregates in their EM diameter, making the use of aggregated proteins for a MW/EMD correlation not an ideal solution in gas‐phase electrophoresis measurements [12].

Nevertheless, the contribution by Bacher et al. 2001 already demonstrated one possible applicability of gas‐phase electrophoresis: the determination of the MW of a proteinaceous compound by determining its EM diameter applying a corresponding correlation function. Hence, especially in times when mass spectrometry struggled to yield MW data for larger compounds, an excellent approximation of the MW for large proteins was achieved. Kapellios et al. compared nES GEMMA derived results for analyte size and MW of several proteins with light scattering results [13]. Other groups targeted other large protein assemblies including inter alia a 3.6 MDa hemoglobin from *Lumbricus terrestris* [14], the 20S proteasome complex [15], a recombinant or PEGylated Van Willebrand Factor [16, 17], or IgG aggregates [18]. In 2007, Kaddis and colleagues published a manuscript in which they applied this approach to determine the MW for large protein complexes like vault macromolecules up to the range of 10 MDa molecular weight. Vaults, being bionanoparticles consisting of two bowl‐like structures yielding an ellipsoid form upon assembly and encapsulating an aqueous interior, could be detected via nES GEMMA and corresponding MW values were calculated applying a protein‐based correlation. However, due to the special analyte structure, already for these macromolecules a deviation in MW between a value based on the EM diameter and a value based on the sum of the individual building blocks was described [19].

## 4 Viruses and Virus‐Like Particle (VLP) MW Determination via nES GEMMA

Besides analysis of proteins with gas‐phase electrophoresis, the suitability of nES GEMMA for the measurement of a human rhinovirus was proven by Allmaier's group [11]. Furthermore, using this system they also studied the tobacco mosaic virus (TMV) and in doing so, found that elongated structures were susceptible for fragmentation during the nES process. Moreover, the obtained corresponding EMD values showed clearly the discrepancy between the EMDs and actual physical size of the analyte [20]. However, spherical bionanoparticles retained their overall shape and immunogenicity as studied via vaccine particles of tick‐borne encephalitis virus (TBEV) [21, 22]. Over the years, the technique matured and more studies applying the gas‐phase electrophoresis for the analysis of other bionanoparticles for instance cowpea mosaic virus (CPMV), rice yellow mottle virus (RYMV) and human adenovirus by Thomas et al. [23] were published. In 2007, Kaddis and colleagues reported the analysis of 4.6 mDa cowpea chlorotic mottle virus (CCMV) [19]. Likewise, phages [24, 25, 26, 27], murine polyomavirus (MPV) [28], or norovirus based virus‐like particles (VLPs) [29] were investigated and research on intact human rhinovirus intensified [30, 31, 32]. Allmaier's Group widened the related research and analyzed gene delivery vectors based on an adeno‐associated virus [33, 34].

Based on their composition, these bionanoparticles either correspond to structures formed from proteins alone (VLPs) or to protein aggregates with encapsulated genomic material in their aqueous interior (viruses and viral vectors). Additionally, e.g., in the case of TBEV, viral capsids were enveloped by a lipid bilayer. Similar to the gas‐phase electrophoretic approach for MW determination of proteins and protein aggregates, soon these analytes as well came to the center of attention of gas‐phase electrophoretic‐based MW determination. In a first instance, Bacher et al. [11] tried to calculate the MW of a human rhinovirus based on nES GEMMA data and their protein‐based EM diameter/MW regression but obtained a value deviating significantly from the expected virus MW (based on the sum of viral building blocks). The authors speculated that this effect resulted from the density of the encapsulated RNA genome and/or the fact that their protein‐based regression was based on data points up to only ~22 nm EM diameter. Mind that the EM diameter of the investigated human rhinovirus was reported to be close to 30 nm and therefore outside the calibrated EM diameter size range. In summary, seemingly, a protein‐based correlation did not yield corresponding results for viral particles.

Despite observed limitations, nES GEMMA data were also used in virus/antibody binding experiments to calculate the number of virus‐bound molecules based on the increase of the particles' EM diameter and application of a protein‐based correlation. Corresponding studies included antibody binding experiments targeting a human rhinovirus [35], or the determination of FAB binding to hepatitis B virus VLP species as published by Bereszczak et al. [36].

As overall consequence, further studies focusing on a development of an EM diameter/MW correlation applicable for characterization of viruses and VLPs were inevitable. However, the availability of well characterized standard material was problematic—at least for a number of bionanoparticle standards the exact MW had to be accessible by MS based measurements in order to generate data for such a correlation. Only recently, especially by the work of the groups of Albert Heck [37, 38, 39, 40, 41], and Martin Jarrold [42, 43, 44] corresponding MS based data became available. Also, lately, new methodologies were introduced to the field, for instance nano‐electromechanical resonator–based mass spectrometry (NEMS MS) based on the work of the group of Christophe Masselon [45, 46]. It was due to these results, that in 2015 it was possible to setup an EM diameter/MW correlation for intact (infectious), non‐enveloped viral particles [47] as well as in 2019 for VLPs [48]. Mind that in both cases, non‐enveloped, spherical particles were targeted. Mind as well that for intact (infectious) viral particles the inherent infectivity of analytes is causing massive experimental problems impeding the further improvement of the described correlation by inclusion of further data points. Due to this reason, the correlation for virus particles available to date only encompasses a limited amount of analytes.

**FIGURE 3:**
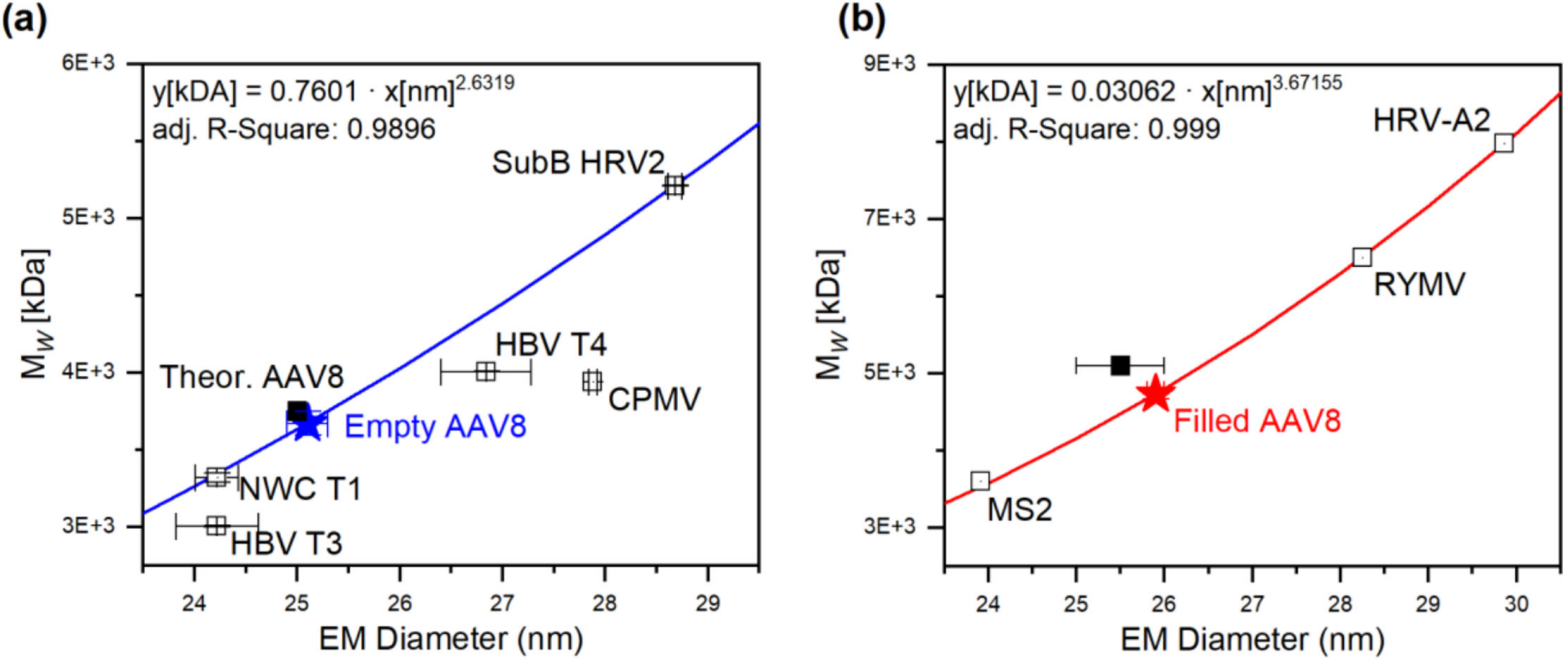
Determination of MW values of empty (A) and filled (B) AAV VLPs based on EM diameter/MW correlations taken from [49] without changes and reproduced under a CC BY 4.0 license.

Based on the correlations available for proteins, viruses, and VLPs, it became evident that in these cases, correlations—targeting different EM diameter and MW regimes—differed from one another (in the case of proteins upon extrapolation). It was therefore concluded that individual correlations needed to be used for each analyte class, i.e., that at least the class of a corresponding analyte needs to be known in order to apply the gas‐phase electrophoresis‐based approach for bionanoparticle MW determination. An analyte class in this context is not so much based on the building blocks of investigated macromolecules—compare e.g., protein aggregates, vaults, and VLPs, all being formed from proteins—but also reflects the three‐dimensional structure of analytes. If such a structure is known, then the technique yields results fast and with little technical prerequisites, as demonstrated by Zoratto and colleagues for the MW determination of an adeno‐associated virus‐based VLP applied for gene‐delivery purposes (see Figure 3). In their work, nES GEMMA derived EM diameter values of empty and filled AAV particles were analyzed, and particle MW values calculated on the basis of corresponding correlations [49].

## 5 Other Analytes

Besides proteins, viruses, and VLPs, the MW assessment, as first demonstrated by Allmaier's Group 2001 [11] with the described technique, was also applied to further analyte classes. Mouradian and colleagues analyzed DNA in 1997 [50] and found a corresponding correlation between the analytes' MW and EM diameter. However, as in the case of Kaufman et al. (1996), the presented data were not used for setting up any corresponding correlation. However, later, using the previously published data points, it became possible to define a corresponding correlation function [51].

In case of polymers, PEGs were measured by Saucy et al. in 2004 [52]. Using company‐provided MW values, a dependence of the analytes' EM diameters on the MW of particles was reported, and a corresponding correlation was introduced. Kemptner and colleagues continued this work in 2010 with PEG derivatives for pharmaceutical applications [53].

In 2007 a combination of two orthogonal techniques, MALDI−TOF−MS and nES GEMMA, targeted PAMAM dendrimers [54] using a protein‐based calibration function for gas‐phase electrophoresis to calculate analyte MW values and to compare obtained results with data obtained from mass spectrometry measurements. There, a significant deviation of MS‐based vs. nES GEMMA‐based MW values was found. However, the observed discrepancies were not further explored for this analyte class.

**FIGURE 4:**
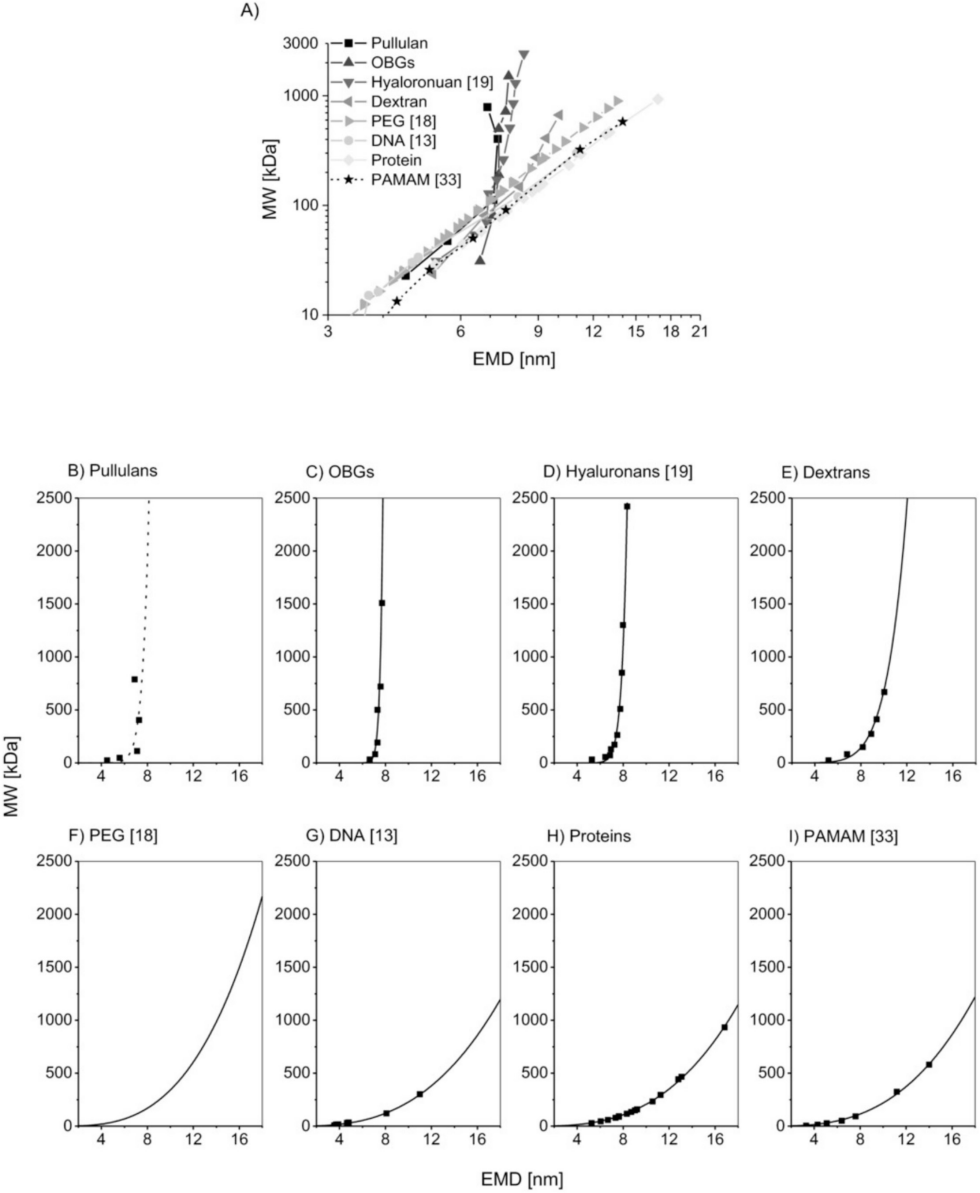
Various EM diameter/MW correlations for linear and branched polysaccharides, polymers, proteins, and DNA, taken from [51] without changes and reproduced under a CC BY 4.0 license.

In 2018, Weiss and colleagues targeted polysaccharides with gas‐phase electrophoresis [51]. In this study, linear polysaccharides like pullulans or oat beta glucans (OBGs) showed a significantly different behavior than branched polysaccharides like dextrans (see Figure 4). For linear polysaccharides, the EM diameter of larger analytes did not increase as expected with the increasing MW. Instead, the EM diameter remained to a certain extent indifferent to changing analyte MW values. In their publication, the authors speculate about various reasons for this effect ranging from e.g., solvation problems, preferential passage of larger analyte moieties through the nES GEMMA system, stabilization of multiple charges on analyte molecules to specific behavior of non‐spherical analytes in the gas phase. Previous investigations of Malm and colleagues showed similar effects as for other linear polysaccharides also for hyaluronans. Nevertheless, nES GEMMA was reported as a method giving “reliable molecular weight estimations” of hyaluronans [55].

Only recently, gas‐phase electrophoresis was also applied to characterize biospecific complexes and to deduce the MW of a proteinaceous transcription factor bound to DNA under manganese mediation [56].

## 6 Conclusions and Outlook

Today's gas‐phase EM analysis of bio‐nanoparticles originated in aerosol science but matures in bioanalytics or pharmaceutical analyses and has also found a number of biotechnological applications, as has been discussed in this contribution.

The instrumentation known as nES GEMMA (also branded MacroIMS, SMPS, or LiquiScan ES) has been widely applied for the determination of MW values for complex analytes in the past decades. However, to allow for high‐accuracy MW determination based on gas‐phase electrophoresis data, additional information about the respective analyte class has to be known, and an appropriate correlation function must be used or developed to correctly interpret the measured spectra. The analyte class in this context does not rely completely on the composition of bionanoparticles in terms of building blocks, but also the structure and shape of macromolecules have to be considered. Protein aggregates, vault molecules, and empty VLPs, for instance—all sharing proteins as building blocks—differ in their corresponding correlation functions. VLPs and viruses of the same origin just differ in the genomic cargo acting as scaffold; likewise, they demand the application of different correlation functions. Table 1 below gives an overview of described correlation functions MW [kDa] = a × EMD [nm]^b^. Correlation functions are listed with declining *b* values, with values close to three indicating spherical particles [19].

**TABLE 1:** Selected published correlation functions MW [kDa] = a × EMD [nm]^b^ for various analyte classes.

	Adj. *R*‐square	*a*	*b*
Pullulans [51]	n. d.	n. d.	n. d.
OBGs [51]	0.930	2E‐26 ± 2E‐25	32.654 ± 6.547
Hyaluronans [51] (fit for data presented in [55])	0.985	3E‐14 ± 6E‐14	18.408 ± 1.150
Dextrans [51]	0.992	7E‐5 ± 7E‐5	6.987 ± 0.459
Viruses [47]	0.999	0.03062	3.67155
PEG [51] (fit for data presented in [52])	1.000	0.244 ± 0.003	3.146 ± 0.004
PAMAM [51] (fit for data presented in [54])	0.997	0.264 ± 0.074	2.919 ± 0.109
Proteins [51]	0.999	0.249 ± 0.021	2.918 ± 0.031
DNA [51] (fit for data presented in [50])	0.998	0.344 ± 0.064	2.821 ± 0.080
VLPs [48]	0.9896	0.7601	2.6319

The authors are aware that instrumentations for gas‐phase electrophoresis had some limitations in the past like (i) radioactive sources for charge equilibration, (ii) the need for manual sample exchange after an individual measurement cycle, or (iii) clogging of nES capillaries by aggregating analytes. However, several of these problems have already been targeted in the last years, significantly improving the instrumental performance. Various sources for bipolar charge equilibration of analytes were investigated and their use optimized [57]. Soft X‐ray‐based charge equilibration was introduced, as was a corona discharge process, either unipolar [58] or bipolar [59] or in a dual spray [60]. Also, the proper manufacturing of capillaries in‐house, which is extremely important for the stable and reproducible nES process, can nowadays be perfectly accomplished based on the work of Tycova et al. [61] making the procedure straightforward, easy, and inexpensive.

Further recent advancement of the instrumental developments has been obtained by applying much higher sheath flow rates inside the DMA units, or making improvements concerning the analyte transfer from the liquid to the gas phase, thereby significantly increasing the resolution of spectra of investigated species, for instance viruses or VLPs [62, 63, 64, 65, 66, 67]. Therefore, it can be expected that the technique of gas‐phase electrophoresis will continue to advance in the following years, focusing e.g., on new analyte classes or its application in real‐time bio‐threat monitoring. The authors are confident that there are still scientific mysteries out there, for which the described technique can be utilized, deepening the understanding of processes on the nanoscale.

## Conflicts of Interest

The authors of this manuscript are also editors of the special issue.

## Data Availability

Data presented in indicated manuscripts was used for this review article.
